# Evaluating an electronic patient-reported outcome monitoring system in patients with prostate cancer in routine clinical care: a prospective observational study

**DOI:** 10.1007/s11136-025-03977-0

**Published:** 2025-04-22

**Authors:** Samuel M. Vorbach, Francesco Sparano, Stefanie Kreissl, David Riedl, Manuel P. Sarcletti, Angela Ginestet, Bernhard Holzner, Ute Ganswindt, Jens Lehmann

**Affiliations:** 1 Department of Radiation Oncology, Medical University of Innsbruck, Innrain 52, AT-6020 Innsbruck, Austria; 2 Data Center and Health Outcomes Research Unit, Italian Group for Adult Haematologic Diseases (GIMEMA), Rome, Italy; 3 Ordensklinikum Linz Elisabethinen, Linz, Austria; 4 Department of Psychiatry, Psychotherapy, Psychosomatics and Medical Psychology, University Hospital of Psychiatry II, Medical University of Innsbruck, Innsbruck, Austria; 5 Evaluation Software Development GmbH, Innsbruck, Austria

**Keywords:** Patient reported outcome measures, Cancer, Electronic patient-reported outcome (ePRO), Radiation therapy

## Abstract

**Purpose:**

This study evaluated the feasibility, acceptability, and clinical utility of an electronic patient-reported outcome (ePRO) system for patients undergoing radiation therapy (RT) for prostate cancer in routine clinical practice.

**Methods:**

A prospective observational study was conducted at the Medical University of Innsbruck among patients receiving RT for primary or recurrent prostate cancer. The ePRO system was designed to capture patient-reported symptoms and health-related quality of life throughout treatment and follow-up, aiming to standardize symptom monitoring and improve continuity of care. Patients completed questionnaires at baseline, during treatment, and in follow-up with feasibility defined as ≥ 75% of patients completing at least 75% of scheduled assessments. Patients’ acceptability was evaluated at baseline and at treatment end using self-developed questionnaires. Healthcare professionals’ (HCPs) perception of clinical utility of the system was measured with a self-developed questionnaire.

**Results:**

Forty patients participated in the study with a median age of 71 years. The ePRO system was feasible, with an average completion rate of 87%, exceeding the feasibility threshold. Patient acceptability was high, with 98% (39/40) expressing willingness to use it regularly. HCPs’ feedback was also positive, with all HCPs (100%, 8/8) reporting usefulness of the system in clinical care and 83% reporting that the system helped them in the clinical assessment of their patients.

**Conclusion:**

The ePRO monitoring system was feasible and well-accepted among both patients and HCPs, demonstrating potential for continued use in routine clinical care. Further efforts are needed to optimize clinical integration and address barriers to ensure equitable access.

**Supplementary Information:**

The online version contains supplementary material available at 10.1007/s11136-025-03977-0.

## Plain English summary

This study was conducted to evaluate a new digital system that helps monitor symptoms and quality of life for men undergoing radiation therapy for prostate cancer. The study aimed to address the need for better ways to track how prostate cancer patients feel during and after their treatment. We wanted to find out if this electronic system would be easy for patients to use and if it would be useful for doctors. Our study included 40 men, mostly over 70 years old, who used the system to complete surveys about their health. We found that most patients could use the system without problems, and nearly all said they were willing to use it regularly as part of their care. Doctors also reported that the system helped them better understand their patients’ symptoms. Our results show that using digital tools like this can potentially help improve the care of prostate cancer patients, though some challenges remain, such as making sure all patients have access to the technology. Overall, this system has the potential to make it easier for doctors and patients to work together to manage symptoms during cancer treatment.

## Background

Today, prostate cancer (PCa) is the second most common cancer in men with over 1.46 million cases worldwide in 2022 [1]. The disease is generally associated with increasing age with highest incidences in men older than 65 years [2]. Radiation therapy (RT) is a widely used and effective treatment for prostate cancer, either as monotherapy or in combination with androgen deprivation therapy (ADT), depending on risk stratification [3–6]. In the past decades, advances in imaging technology and radiation techniques have led to improved target coverage while minimizing toxicity to surrounding healthy tissues [7, 8]. Still, RT is associated with relevant toxicities including acute, late, and permanent urinary, bowel and sexual dysfunction, which may adversely affect patients` overall health-related quality of life (HRQoL) [9, 10].

Fortunately, many patients who undergo RT for prostate cancer regain HRQoL levels comparable to that of the general population [10]. Nevertheless, it remains crucial to identify those affected by acute or long-term treatment sequelae early on to enable timely interventions. Monitoring symptom burden during or after RT presents unique challenges, as the treatment is typically administered in an outpatient setting with less frequent clinical visits compared to systemic therapies. Additionally, the high volume of patients often necessitates treatment at various times, sometimes in the evening, with patients frequently being seen by different physicians who may not be fully aware of their HRQoL history. This can lead to a lack of continuity in care.

To address these challenges, the use of Patient-Reported Outcome Measures (PROMs) has emerged as a valuable tool. PROMs provide a systematic way to capture patients’ symptoms, side effects, and overall quality of life, offering a real-time and patient-centered perspective on their health status, which can be made available to all treating physicians [11]. When integrated into clinical care, PROMs can enhance patient outcomes by enabling timely interventions for symptom deterioration [11–14] and use of PROMs has shown to benefit clinical endpoints in recent international meta analyses [15, 16]. Despite advancements in the integration into routine oncology clinical practice, large-scale implementations and broad use of PROMs remain limited in clinical care worldwide [17–19]. For example, a 2023 survey of German-speaking radiation oncologists found that 83.7% of participants were unaware of PROs [20]. This highlights the need for optimized implementation strategies both globally and regionally.

While some pilot projects have integrated PROMs into clinical care during radiation therapy, wider implementation is still limited [21, 22]. Integrating PROMs into routine care poses significant challenges, including workflow integration, digital literacy (if PROMs are assessed electronically), and resource allocation [23, 24]. If PROMs are assessed electronically, such systems are often referred to as electronic PRO (ePRO) systems. A recent survey among radiation oncology departments in Germany, Austria, and Switzerland revealed that only 9% of departments use ePRO systems as part of their care [25].

Additional research is crucial to understand patient engagement with ePRO systems and to inform care strategies for patients with PCa receiving radiation therapy. Insights into patient perspectives on ePRO systems will be critical to developing effective and sustainable models, ultimately enhancing patient outcomes and satisfaction with care. Thus, the aim of this study was to develop and test an ePRO system for patients receiving RT for PCa. Integrating electronic symptom monitoring into clinical care potentially allows for real-time symptom tracking, enabling timely interventions and more personalized patient management during and after radiotherapy [15, 16]. By systematically collecting patient-reported data, PROMs can enhance communication between patients and healthcare providers, helping to bridge gaps in care and improving overall treatment quality. We aimed to evaluate the feasibility and acceptability of a locally developed ePRO system from both the patient and the healthcare professional (HCP) perspective in order to successfully establish the system in clinical routine.

## Methods

### Study design and patients

**Fig. 1:**
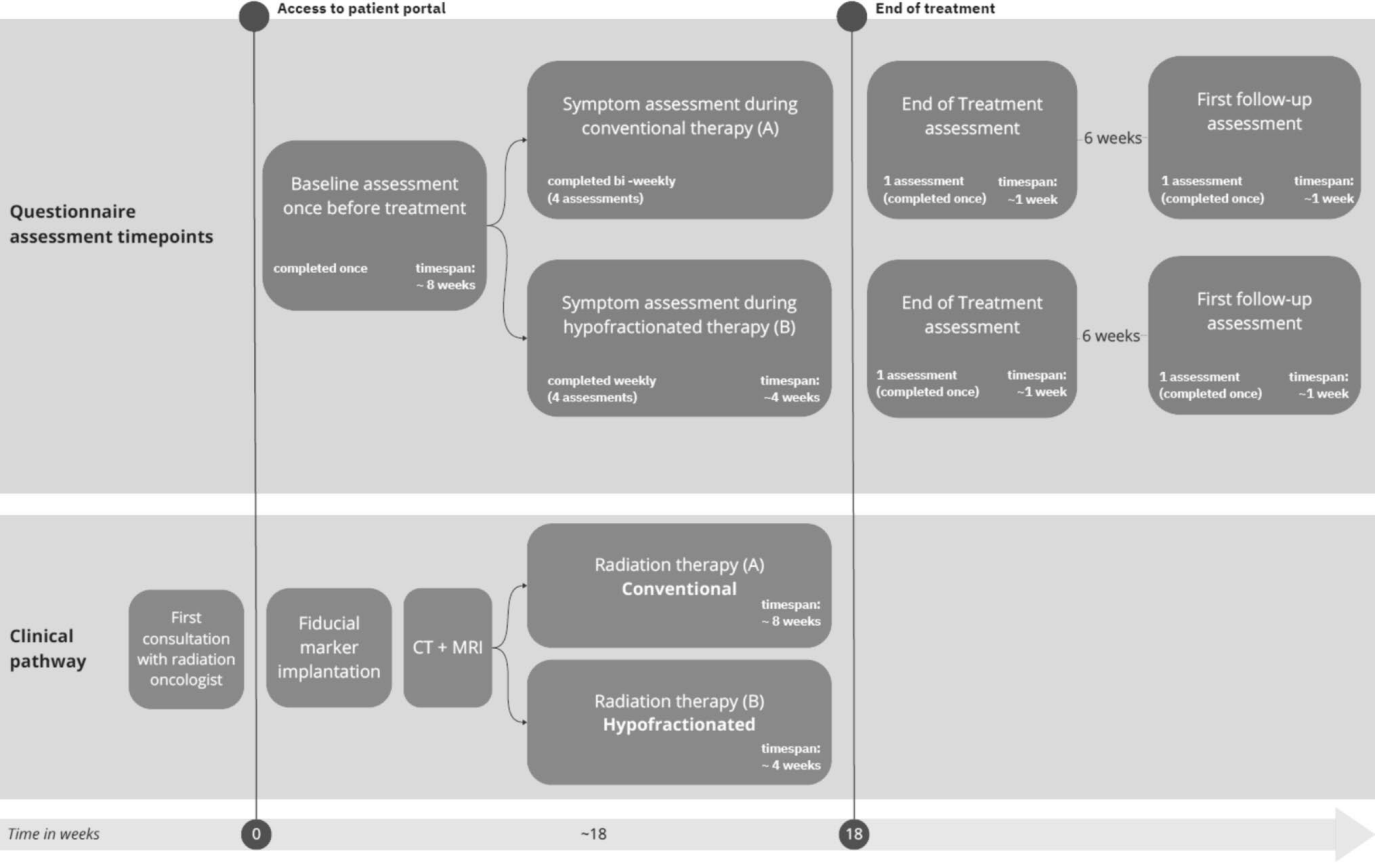
Clinical flowchart and overview of assessments. Illustration of the questionnaire assessment time points (green) within the radiation oncology workflow (blue). Shown are conventional (**A**) and hypofractionated (**B**) radiation therapy

We conducted a prospective observational study consecutively recruiting patients at the Department of Radiation Oncology at the Medical University of Innsbruck. The study is reported according to the Strengthening the Reporting of Observational Studies in Epidemiology (STROBE) guidelines [26] (Supplementary Material 1). The study was approved by the institutional review board of the Medical University of Innsbruck (EC No. approval: 1265/2023) and the study was conducted in accordance with the declaration of Helsinki (1964). This observational study was designed to evaluate the feasibility and acceptance of an ePRO system among patients undergoing radiotherapy for prostate cancer. Patients were enrolled before the start of radiotherapy and followed throughout their treatment and six weeks post-radiotherapy. The methodological approach involved longitudinal data collection through an ePRO system, with assessments conducted at baseline (pre-treatment), during radiotherapy, and at follow-up (see section description of the ePRO monitoring program and Fig. 1). Feasibility was defined based on patient adherence to completing scheduled assessments, while acceptability was evaluated through two self-developed questionnaires. Additionally, HCPs’ perceptions of the ePRO system’s clinical utility were assessed. The findings aimed to determine the practicality of integrating electronically assessed PROMs into routine clinical practice for real-time symptom management and patient-centered care.

Given the study’s primary focus on feasibility and acceptability rather than hypothesis testing, no formal sample size calculation was performed. Instead, we used a convenience sample of 40 patients, who we were able to recruit within a reasonable timeframe and which was deemed sufficient to evaluate feasibility and acceptability.

Patients were approached consecutively and considered eligible if they (1) received RT for primary PCa or biochemical/local recurrence, and (2) had sufficient German language skills to complete the questionnaires. All participants provided written informed consent before recruitment.

### Description of the electronic patient-reported outcome system

The ePRO system was developed and integrated into the clinical workflow at the Department of Radiation Oncology, Medical University of Innsbruck, to provide continuous monitoring of patients both during and after RT. The program was built based on the Computer-based Health Evaluation System (CHES) [27], an established platform designed for the digital collection and analysis of HRQoL data.

In short, the workflow for prostate cancer patients in our department consists of an initial consultation, followed by fiducial marker implantation within the next three weeks. Two to four weeks after the fiducial marker implantation, which is not mandatory for all patients, a CT and MRI scan are performed for treatment planning. The actual treatment consists of five fractions per week for a total of 4 to 8 weeks. We integrated the ePRO system into this process (see Fig. 1). During their first visit at the department, patients were given access to the ePRO system (patient portal) by administrative staff. With the login credentials, patients were able to log into the portal and review information about the department, their treatment, complete questionnaires, and review their PRO scores (see Supplementary Material 2). Patients were instructed to complete questionnaires both during treatment and aftercare by their treating physician and received automated reminders (SMS or email) every time new questionnaires were available. As soon as new questionnaires became available, the patients could complete these on any electronic device. If patients had difficulty completing the questionnaires or did not have access to the internet, they could complete the questionnaires on a tablet within the department, if necessary with support from the administrative staff. A nurse was assigned to call patients who had not completed their follow-up questionnaires; however, this measure was implemented during the course of the study and hence not available to all patients in the study. Figure 1 shows the assessment and treatment schedule. Integration into clinical care was a central feature of the ePRO program. Data from the questionnaires were automatically uploaded to a secure database, where HCPs could review the results in real-time. The intended clinical use—recording HRQOL as well as symptom monitoring in patients undergoing radiotherapy, compared to their baseline before therapy, as well as tracking symptoms during follow-up—was explained to the treating physicians by the study team during an initial presentation. More details on the onboarding process are provided in Supplementary Materials 7.

To ensure the selection of the most appropriate questionnaires for monitoring HRQOL, we employed a consensus-driven approach. A panel of five radiation oncologists and two PRO experts reviewed available PROMs, considering their clinical relevance, previous validation in prostate cancer populations, and recommendations from standard outcome sets [28, 29]. Based on this expert consensus, we selected the EORTC QLQ-C30 [30], EPIC-26 [31], and the Hornheider Screening Instrument [32].

Complete versions of these questionnaires were administered at baseline, end of therapy, and during follow-up assessments to provide a thorough evaluation of patient outcomes over time. The Hornheider Screening Instrument was administered once at baseline. During RT, a subset of 21 items from the QLQ-C30 and the EORTC Item Library [33], focusing on treatment-associated symptoms, was used to minimize patient burden while maintaining the assessment’s focus on critical symptoms. More information on how questionnaires and items were selected along with thresholds for clinical interpretation are given in Supplementary Materials 3.

In summary, during the study, patients had the following assessment time points: baseline assessment, multiple symptom assessments during therapy (between 3 to 5 depending on their treatment schedule), end of treatment assessment, and a follow-up assessment 6 weeks after the end of their treatment.

### Study outcomes and statistical analyses

Primary outcomes of this study were feasibility and patients’ acceptability of the ePRO system. Secondary outcome was HCPs’ perception of clinical utility of the system. We considered *feasibility* as demonstrated if ≥ 75% of patients completed ePRO questionnaires in at least 75% of scheduled assessments. *Patients*’* acceptability* was measured at baseline and at the end of treatment using two self-developed questionnaires. At baseline, patients completed a 6-item questionnaire (including one free-text answer for further comments or suggestions) focused on technical feasibility and problems with completion. At the end of treatment, they completed a 4-item questionnaire focusing on the acceptability of the assessment, as well as two open-ended questions for further barriers or comments. Both questionnaires are listed as Supplementary Materials 6.

To measure c*linical utility* of the system, an online questionnaire was administered to HCPs at the end of treatment. HCPs rated, on a 5-point Likert scale (ranging from “Not at all” to “Very much”), 8 items covering aspects such as the relevance of the ePRO questionnaires to their work, the ease of use, and the utility of the ePRO system in their clinical routine.

We report descriptive analyses only for both patients and HCPs. All analyses were conducted with SPSS (version 29.0, IBM).

## Results

Between 27.09.2023 and 12.01.2024, we approached 46 patients who met the inclusion criteria and 40 patients (87.0%) consented to participate in the study. Patients who refused participation did not see benefits to using the ePRO system (n = 6, 13.0%). The median age at the start of RT was 71 years (range 48–86, Table 1). Most patients had an International Society of Urological Pathology (ISUP) score of 1 or 2 (67.5%) at initial diagnosis. 19 of 40 patients (45%) received salvage RT for biochemical recurrence (BCR) after radical prostatectomy (RPE). Median time from RPE to BCR was 24 months (range 5–103). The remaining 21 patients received RT as initial treatment for their prostate cancer.

**Table 1:** Patient sociodemographic and clinical information

Variable	N = 40^1^
Age (years)	71 (48–86)
ISUP Score at initial diagnosis	
1	6 (15.0%)
2	21 (52.5%)
3	6 (15.0%)
4	1 (2.5%)
5	6 (15.0%)
Radiotherapy	
Primary Radiotherapy	21 (55.0%)
Salvage Radiotherapy for biochemical / local recurrence	19 (45.0%)
Concomitant ADT	
Yes	24 (60.0%)
No	16 (40.0%)
PSA at treatment start (ng/ml)	3.10 (0.14–25.06)
Radiation therapy	
Conventional	29 (72.5%)
Hypofractionated	11 (27.5%)
ECOG Performance Status	
0	27 (67.5%)
1	12 (30.0%)
2	1 (2.5%)
Comorbidities	
≥ 1	30 (75.0%)
0	10 (25.0%)
Education	
Compulsory school with apprenticeship	20 (50.0%)
High school	9 (22.5%)
University degree	6 (15.0%)
Compulsory school without apprenticeship	4 (10.0%)
Other/unknown	1 (2.5%)
Living situation	
Living with partner/own family/children	35 (87.5%)
Living alone	5 (12.5%)
Marital status	
Married, cohabitation, partnership	33 (82.5%)
Divorced, separated, widowed	6 (15.0%)
Single	1 (2.5%)
Profession	
Retired	29 (72.5%)
Fully employed	6 (15.0%)
Temporary pension / ongoing application	2 (5.0%)
Unemployed	1 (2.5%)
Part-time employee	1 (2.5%)
Sick leave (longer than three months)	1 (2.5%)
Are you familiar with the use of the internet?	
Yes	34 (85.0%)
No	6 (15.0%)
Which internet devices do you use? (more than one option could be chosen)	
Smartphone/Mobile phone	25 (62.5%)
Desktop/PC	22 (56.4%)
Laptop	15 (37.5%)
Tablet	6 (15.0%)
No devices used	6 (15.0%)
How often do you use the internet?	
Several times a day	17 (42.5%)
Once a day	8 (20.0%)
1–3 times a week	8 (20.0%)
Not specified	5 (12.5%)
1 × per month	1 (2.5%)
Rarer	1 (2.5%)
Do you own a smartphone?	
Yes, frequently used	19 (47.5%)
Yes, rarely used	12 (30.0%)
Never	9 (22.5%)
Do you own a tablet?	
Yes, frequently used	5 (12.5%)
Yes, rarely used	9 (22.5%)
No	26 (65.0%)
How often do you use email?	
Frequently	28 (70.0%)
Rarely	12 (30.0%)
Not at all	0 (0%)

^1^Median (range); n (%)

Sixty percent of all patients (n = 24) had concomitant ADT. Median PSA at the treatment start was 3.10 ng/mL. Twenty-nine out of 40 patients (72.5%) were treated with conventional RT, and eleven (27.5%) with moderate hypofractionated RT. Most patients had an ECOG Performance Status of 0 (n = 27, 67.5%) and 75.0% of patients (n = 30) had at least one comorbidity. The majority of patients (n = 35, 87.5%) lived with others (a partner or a member of the family) and only 5 (12.5%) lived alone. Twenty-nine patients (72.5%) were retired at the time of study entry, and six (15.0%) were fully employed. Most of the patients (n = 34, 85.0%) were familiar with using the internet, and almost half (n = 17, 42.5%) used it several times a day. Six patients (15.0%) reported not to use any internet-ready device.

### Feasibility of completing ePRO assessments

Completion rates for scheduled assessments were high for almost all patients. The average completion rate was 87.2%. Our threshold for feasibility with regard to completing scheduled questionnaires was reached (≥ 75% of patients completing ≥ 75% of scheduled questionnaires) and only 5 patients (12.5%) had a completion rate below 75%. The average completion rate before and during treatment was 89.9%, while completion for the follow-up assessments was somewhat lower at 72.5%. A total of 11 patients (17.5%) did not complete the follow-up assessment. Initially, during the assessment period and for the first 6 of these patients, there was no nurse to call the patient if the assessment was not completed and remind them to complete the questionnaire. One patient did not complete the remote follow-up due to lack of internet access and 3 patients did not complete the follow-up as they had previously completed the questionnaires in the hospital using the provided tablet and did not have an own device.

### Acceptability of the ePRO system and assessments

**Fig. 2:**
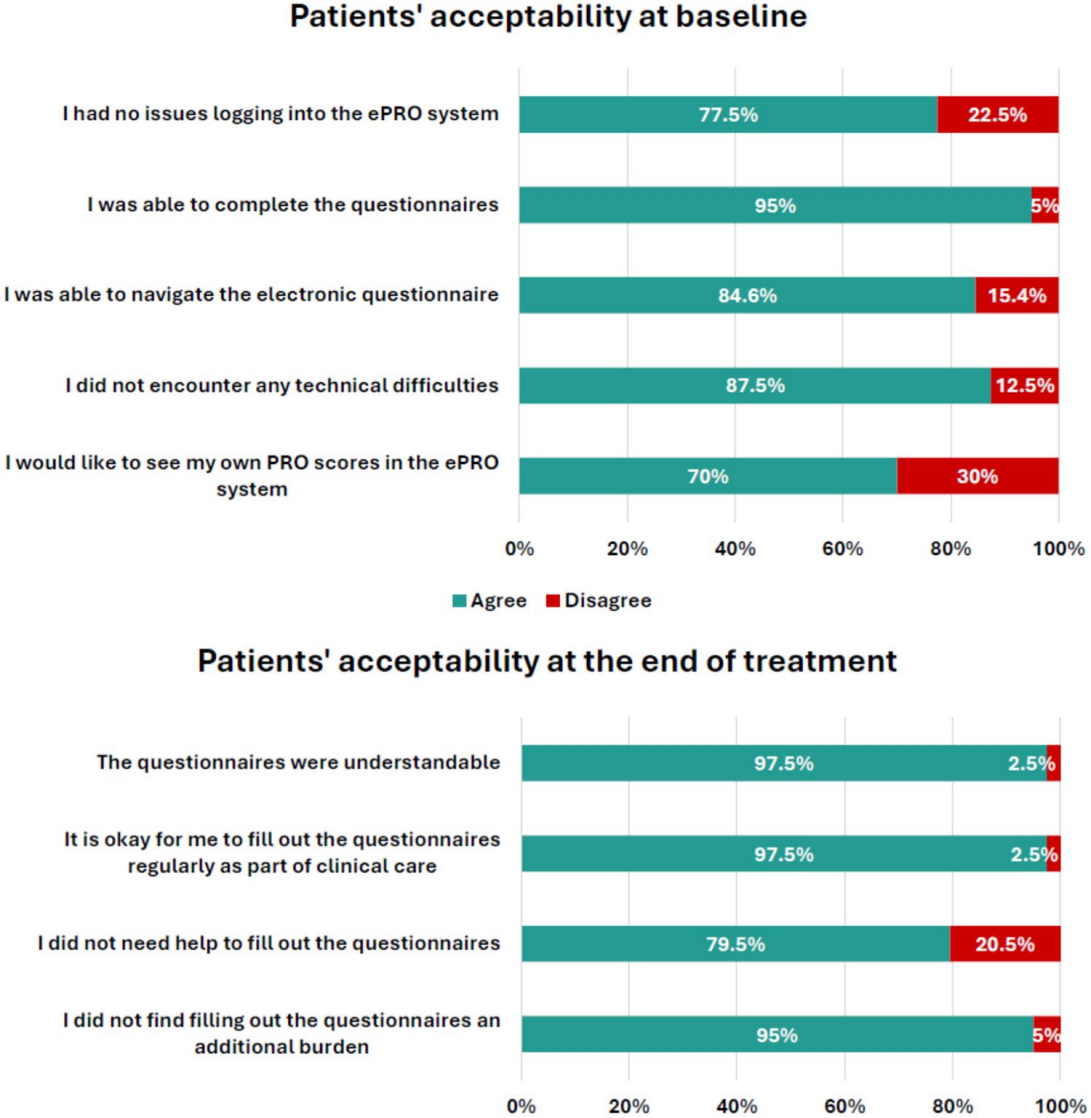
Patient answers to technical feasibility questions and acceptability questions. For graphical purposes, the questions and answers were paraphrased. The original wording is reported in the appendix

Of the 40 patients who participated in this study, all completed the acceptability survey at baseline, as well as at the end of treatment. The survey results indicate that, overall, patients were satisfied with the ePRO system and found it easy to use (Fig. 2).

At baseline, most of the patients (95.0%, 38/40, Supplementary Material 4) indicated that they were able to complete the electronic questionnaires, and 84.6% (33/39) were able to navigate the questionnaires (i.e. forward, back, finish) without issues. The majority of patients (87.5%, 35/40) encountered no technical difficulties using the system, and 77.5% (31/40) reported no issues logging into the ePRO system. More than two-thirds of the patients (70.0%, 28/40) confirmed that they would like to see their own results after completing the questionnaires. The most frequently reported reasons (optional free text) were interest/curiosity in seeing their PRO scores and being informed about the progress of their disease.

Positive feedback was also registered at the end of treatment. Almost all patients (97.5%, 39/40) found the selected questionnaires understandable and indicated their willingness to regularly fill out the questionnaires as part of their clinical care. The majority of patients (79.5%, 31/39) did not need help completing the questionnaires, and 95.0% (38/40) did not find the questionnaires to be an additional burden. When asked about obstacles encountered during the electronic completion of questionnaires, the few patients who optionally added a comment most frequently mentioned receiving help by a family member as a way to overcome any obstacles encountered when using the platform (data not shown).

To evaluate the clinical utility of the ePRO system in routine practice, we surveyed all HCPs utilizing the system. All HCPs who interacted with the system completed the survey, including five physicians (62.5%), two administrators (25.0%) responsible for granting patient access to the portal, and one psycho-oncologist (12.5%). The administrative staff, both with at least 3 years of professional experience, stated that using the system entailed an additional workload of 5 to 20 min per day and that the training they had received was understandable and sufficient.

When asked about the training received before using the ePRO system, almost all of the physicians and the psycho-oncologists (83.3%, 5/6) found it “very much” or “quite a bit” understandable and sufficient. They would “very much” (66.7%, 4/6) or “quite a bit” (16.7%, 1/6) recommend this system to other colleagues. Further information and results by professional group are shown in the appendix (Supplementary Material 5).

**Fig. 3:**
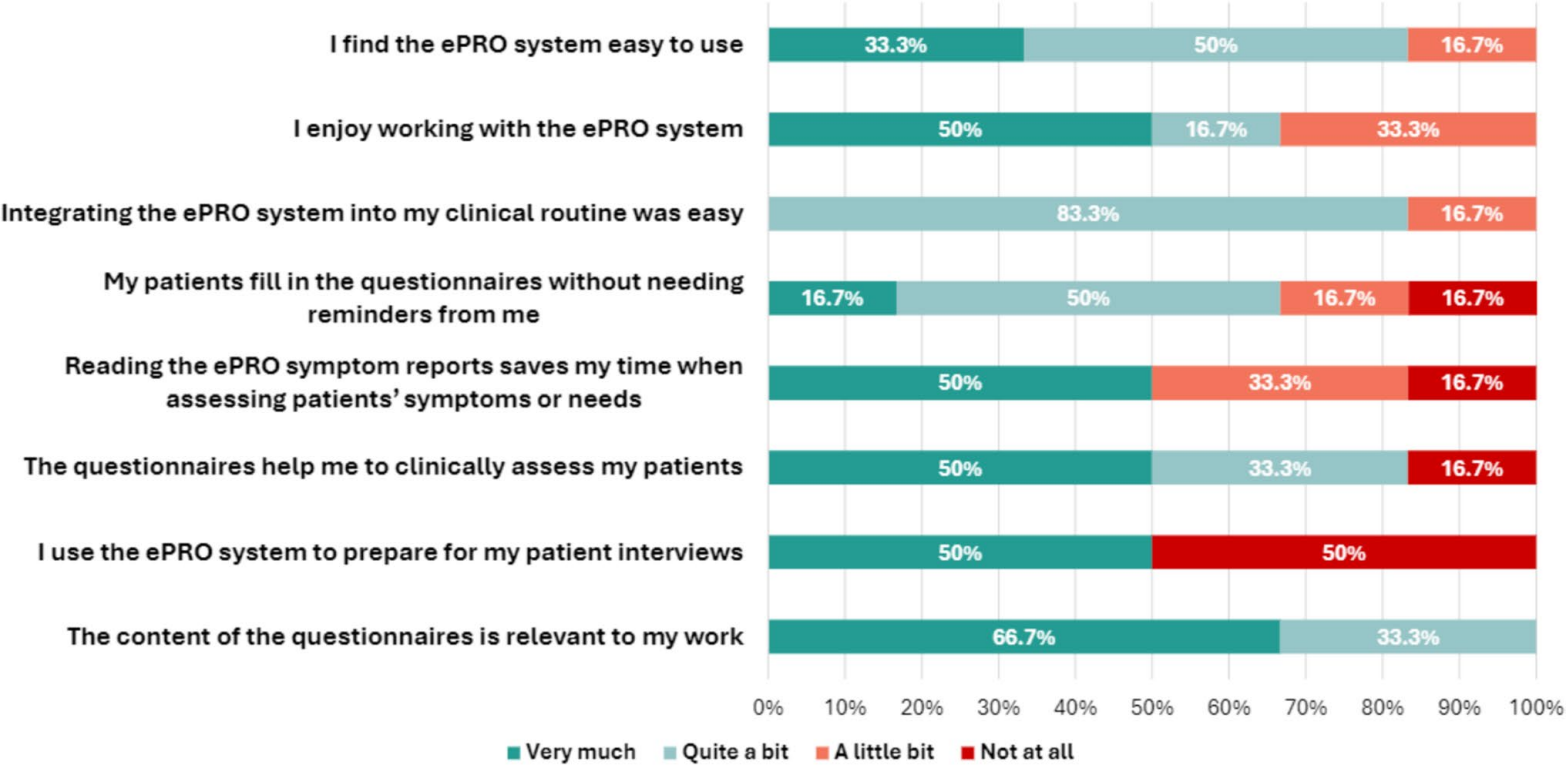
Physicians’ and psycho-oncologist’s acceptability answers

Additional questions specifically addressed to physicians or psycho-oncologists (n = 6) showed that, overall, they found the ePRO system useful (Fig. 3). Five of them (83.3%) found that the ePRO system was “very much” or “quite a bit” easy to use, and five (83.3%) found it “quite a bit” easy to integrate it in their clinical routine. All of them (100.0%, 6/6) deemed the content of the questionnaires “very much” or “quite a bit” relevant for their work, and almost all (83.3%, 5/6) reported that the questionnaires “very much” or “quite a bit” help them to clinically assess their patients.

## Discussion

In this study, we evaluated the feasibility, acceptability, and clinical utility of an ePRO system in patients undergoing RT for prostate cancer. The high completion rates and positive feedback from both patients and HCPs highlight the potential of ePRO systems to enhance symptom monitoring and improve patient care in routine clinical settings.

Our findings demonstrate that the ePRO system was feasible to use in clinical practice, with an average completion rate of 87.2% for scheduled assessments, surpassing our feasibility threshold. This suggests that patients are willing and able to engage with electronic systems to regularly report their symptoms. This is in line with previously published data indicating high adherence rates for ePRO systems in oncology settings. For example, patient compliance with weekly symptom reporting observed by Basch et al. was 91.5% [34], while weekly adherence reported by Denis et al. [35] was 75%. Our results align with previous studies conducted in the context of PCa. In a single-arm pilot trial exploring the feasibility and acceptability of an ePRO system, 86% of patients completed at least 60% of weekly questionnaires [22]. Another feasibility study conducted in PCa patients receiving palliative systemic treatment showed an 89% uptake [36], which is consistent with our study, with 87% of invited patients consenting to participate in the study.

Patient acceptability was also notably high in our study, with most of the patients finding the system easy to use and expressing willingness to use it regularly as part of their care. This positive feedback aligns with findings from other studies that have shown high levels of patient satisfaction with ePRO systems in oncology settings. For example, in the PRO-TECT trial, the majority of patients found the online system easy to use, understandable, and valuable for quality of care [37]. A systematic review examining the features of five ePRO systems for monitoring immune-related adverse events across different tumor types found high rates of patient acceptability [38]. However, the same authors noted heterogeneity in the definition and measurements of acceptability. The low rate of technical difficulties encountered by patients, the system’s ease of use, along with features implemented to facilitate users’ experience, such as sending regular reminders, may have contributed to the high acceptability rates shown in our study. This is particularly important considering that PCa is generally diagnosed in older men, and not all of whom may be familiar with technology.

Despite the promising findings, implementing ePRO systems in routine care presents several challenges. One challenge is ensuring that all patients have access to the necessary technology to complete ePRO assessments. In our population, 15% of patients reported not to be familiar with internet use or not using any internet device. Not having internet-ready devices was the reason why some PCa patients were lost to follow-up. This highlights the need for providing technological support or alternative modes of completion to patients to ensure equitable access to ePRO systems. Furthermore, 20% of the patients in this study needed help, mostly from their family, to complete the ePRO questionnaires. Family support to facilitate digital access was also previously noted [36, 39]. Taken together, these data underscore the need to implement strategies to provide additional support to patients with less technical abilities, for example from a member of the care team, and to consider all the potential barriers, to avoid disparities in care and ensure that PRO assessment is as inclusive as possible [23, 40]. Previous evidence showed that clinical benefits associated with ePRO systems might be even greater in patients without computer experience [13].

Another finding from our study is the positive feedback from HCPs, indicating that the ePRO system is relevant and useful for their routine clinical practice. Results from the HCPs survey suggest that the adoption of an ePRO system can facilitate clinicians’ work and communication with their patients. Indeed, all HCPs deemed the content of the questionnaires relevant for their work and most of them reported that the questionnaires help them to clinically assess their patients. These results are similar to those found, for example, in the PRO-TECT trial, where almost 65% of the oncologists reported to use PROs information to make treatment decisions [37]. However, integrating ePRO data into existing clinical workflows might still represent an important challenge. Although most HCPs in this study deemed the system clinically useful, the additional workload associated with using the system needs to be considered. Indeed, half of HCPs reported that reading the ePRO symptoms report, did not save time (or did it only a little bit) when assessing their patients. Also, half of them reported not to use ePROs to prepare for the interviews with their patients, and this may also be possibly explained by the lack of time. Thus, strategies to streamline the integration of ePRO data into clinical workflows and minimize additional workload are essential for the successful adoption of these systems. A role may be played for example by oncology nurses, as recommended by the recent ESMO Clinical Practice Guideline [11], who may act as first responders to PRO alerts and help manage symptoms monitoring. Another area for improvement is to optimize alert notifications and thresholds in the system to reduce alert fatigue and distinguish urgent from non-urgent cases [41].

## Limitations

Our study has some limitations. First, the single-center design and specific application to patients with PCa receiving RT limit the generalizability of our findings. At the same time, this approach also allowed us to tailor the ePRO system to patients’ and HCPs’ specific needs, which likely contributed to their use and satisfaction. Second, although we tried to also include patients with limited technical capabilities and access, some patients were unable to participate in the remote follow up assessments or in the monitoring as a whole. Additionally, a flexible approach that considers both patients’ and HCPs’ preferences for receiving alerts could also be explored. Indeed, a recent trial showed that sending alerts directly to the patients is as equally effective as sending them to HCPs in improving HRQoL, while being less burdersome for HCPs [42].

## Conclusion

Our study demonstrates that our ePRO system was both feasible and acceptable for use in patients undergoing radiation therapy for prostate cancer, with high completion rates and positive feedback from both patients and HCPs. While the system shows promise for enhancing symptom monitoring and improving patient care, challenges remain in ensuring equitable access and integrating PRO data into clinical workflows without adding to the workload of HCPs. Future efforts should focus on optimizing system usability, providing additional support to patients with limited technical skills, and developing strategies to streamline the integration of ePRO systems into routine clinical practice.

## Supplementary Information

Below is the link to the electronic supplementary material.

Supplementary file1 (PDF 1875 kb)

## Data Availability

The data from this article cannot be shared due to data protection laws and the informed consent not covering sharing the data used in the study. Anonymized (aggregated) metadata can be obtained by the corresponding author upon reasonable request.
