# Supplementary material for: Evaluating an electronic patient-reported outcome monitoring system in patients with prostate cancer in routine clinical care: a prospective observational study
Source: Qual Life Res. 2025 Apr 22;34(7):2085–96. doi: 10.1007/s11136-025-03977-0 (PMC12182459; doi:10.1007/s11136-025-03977-0)
Supplement: Supplementary file 1 — Supplementary file1 (PDF 1875 kb) [file 11136_2025_3977_MOESM1_ESM.pdf]

Supplementary Materials

Supplementary Material 1: STROBE Statement

STROBE Statement—checklist of items that should be included in reports of observational studies

	Item No	Recommendation	Reported where? (if not in fulltext, information is provided here)
**Title and abstract**	1	(*a*) Indicate the study's design with a commonly used term in the title or the abstract	Title ("a prospective observational study)
		(*b*) Provide in the abstract an informative and balanced summary of what was done and what was found	Abstract
**Introduction**			
Background/rationale	2	Explain the scientific background and rationale for the investigation being reported	Section: Background
Objectives	3	State specific objectives, including any prespecified hypotheses	Section: Background – last paragraph
**Methods**			
Study design	4	Present key elements of study design early in the paper	First section of methods: "Study design and patients"
Setting	5	Describe the setting, locations, and relevant dates, including periods of recruitment, exposure, follow-up, and data collection	Methods sections: "Description of the electronic patient-reported outcome monitoring program" & Supplementary Materials 3: Questionnaires used and item and threshold selection process & results (first paragraph)
Participants	6	**(*a*) *Cohort study*—Give the eligibility criteria, and the sources**	Methods: Study design and patients & Description of the electronic

		**and methods of selection of participants. Describe methods of follow-up**	patient-reported outcome monitoring program & Study outcomes
		(*b*) *Cohort study*—For matched studies, give matching criteria and number of exposed and unexposed	Not applicable
Variables	7	Clearly define all outcomes, exposures, predictors, potential confounders, and effect modifiers. Give diagnostic criteria, if applicable	Methods: Study outcomes
Data sources/ measurement	8*	For each variable of interest, give sources of data and details of methods of assessment (measurement). Describe comparability of assessment methods if there is more than one group	Methods: Study outcomes & Supplementary Materials 3: Questionnaires used and item and threshold selection process
Bias	9	Describe any efforts to address potential sources of bias	Not applicable. As the study was conducted in routine clinical care and recruitment was conducted consecutively, we only expect bias in terms of technical capacities (which is discussed in the limitations section).
Study size	10	Explain how the study size was arrived at	Methods: Study design and patients
Quantitative variables	11	Explain how quantitative variables were handled in the analyses. If applicable, describe which groupings were chosen and why	Methods: Study outcomes and statistical analyses

Statistical methods	12	(*a*) Describe all statistical methods, including those used to control for confounding	Methods: Study outcomes and statistical analyses
		(*b*) Describe any methods used to examine subgroups and interactions	Not applicable
		(*c*) Explain how missing data were addressed	Missing data are considered a result and are reported in the section “Feasibility of completing ePRO assessments”
		(*d*) *Cohort study*—If applicable, explain how loss to follow-up was addressed	Loss to follow-up was considered a result and is reported in the section “Feasibility of completing ePRO assessments”
		(*e*) Describe any sensitivity analyses	Not applicable.

**Results**			**Reported where? (if not in fulltext, information is provided here)**
Participants	13*	(a) Report numbers of individuals at each stage of study—eg numbers potentially eligible, examined for eligibility, confirmed eligible, included in the study, completing follow-up, and analysed	Results: First paragraph
		(b) Give reasons for non-participation at each stage	Results: First paragraph
		(c) Consider use of a flow diagram	Not applicable.
Descriptive data	14*	(a) Give characteristics of study participants (eg demographic, clinical, social) and information on exposures and potential confounders	Results: Table 1

		(b) Indicate number of participants with missing data for each variable of interest	Results: Feasibility of completing ePRO assessments & Acceptability of the ePRO assessments/system
		(c) *Cohort study*—Summarise follow-up time (eg, average and total amount)	Not applicable.
Outcome data	15*	*Cohort study*—Report numbers of outcome events or summary measures over time	Results: Feasibility of completing ePRO assessments & Acceptability of the ePRO assessments/system
Main results	16	(*a*) Give unadjusted estimates and, if applicable, confounder-adjusted estimates and their precision (eg, 95% confidence interval). Make clear which confounders were adjusted for and why they were included	Results: Feasibility of completing ePRO assessments & Acceptability of the ePRO assessments/system<br><br>No adjustment was done.
		(*b*) Report category boundaries when continuous variables were categorized	Not applicable.
		(*c*) If relevant, consider translating estimates of relative risk into absolute risk for a meaningful time period	Not applicable.
Other analyses	17	Report other analyses done—eg analyses of subgroups and interactions, and sensitivity analyses	Not applicable.
**Discussion**			
Key results	18	Summarise key results with reference to study objectives	Discussion: First paragraph
Limitations	19	Discuss limitations of the study, taking into account sources of potential bias or imprecision. Discuss both direction and magnitude of any potential bias	Discussion: Limitations
Interpretation	20	Give a cautious overall interpretation of results considering objectives, limitations, multiplicity	Discussion

		of analyses, results from similar studies, and other relevant evidence	
Generalisability	21	Discuss the generalisability (external validity) of the study results	Discussion: Limitations
**Other information**			
Funding	22	Give the source of funding and the role of the funders for the present study and, if applicable, for the original study on which the present article is based	Not applicable. No funding was received

*Give information separately for cases and controls in case-control studies and, if applicable, for exposed and unexposed groups in cohort and cross-sectional studies.

Note: An Explanation and Elaboration article discusses each checklist item and gives methodological background and published examples of transparent reporting. The STROBE checklist is best used in conjunction with this article (freely available on the Web sites of PLoS Medicine at http://www.plosmedicine.org/, Annals of Internal Medicine at http://www.annals.org/, and Epidemiology at http://www.epidem.com/). Information on the STROBE Initiative is available at www.strobe-statement.org.

Supplementary Material 2: ePRO platform screenshots [original in German language, with descriptions]

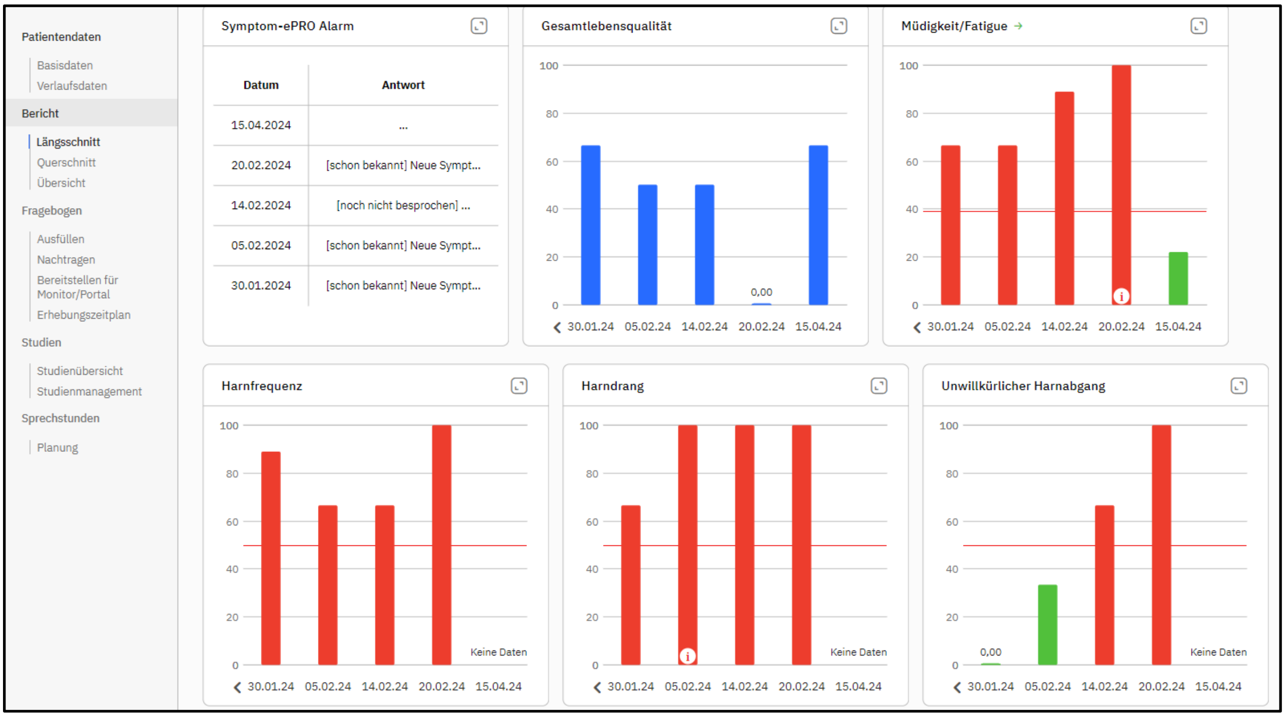

Screenshot 1. This screenshot shows an example of how questionnaire results are displayed for healthcare professionals. The domains in this screenshot include the domains global quality of life (top row, middle), fatigue (top row, right side), and urinary symptoms (bottom row).

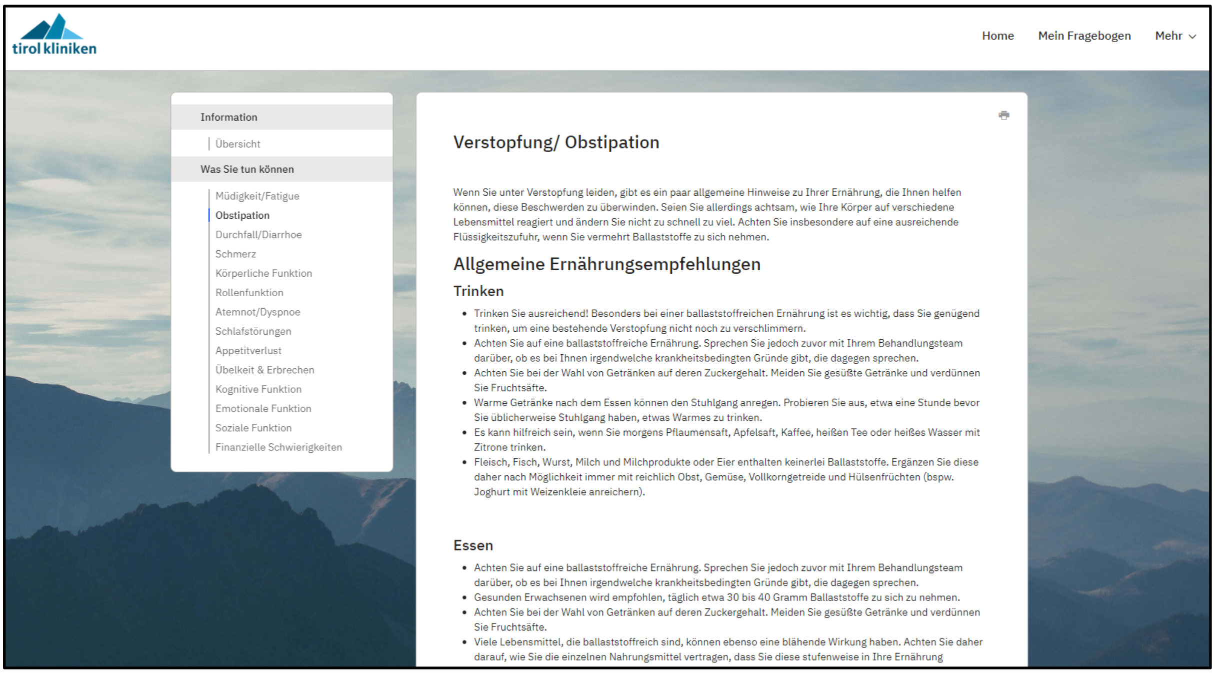

Screenshot 2. This screenshot shows an example of tailored self-management advice in the patient portal. This advice is displayed to patients if they have problems in a specific domain. In this case, information on obstipation is shown containing dietary advice.

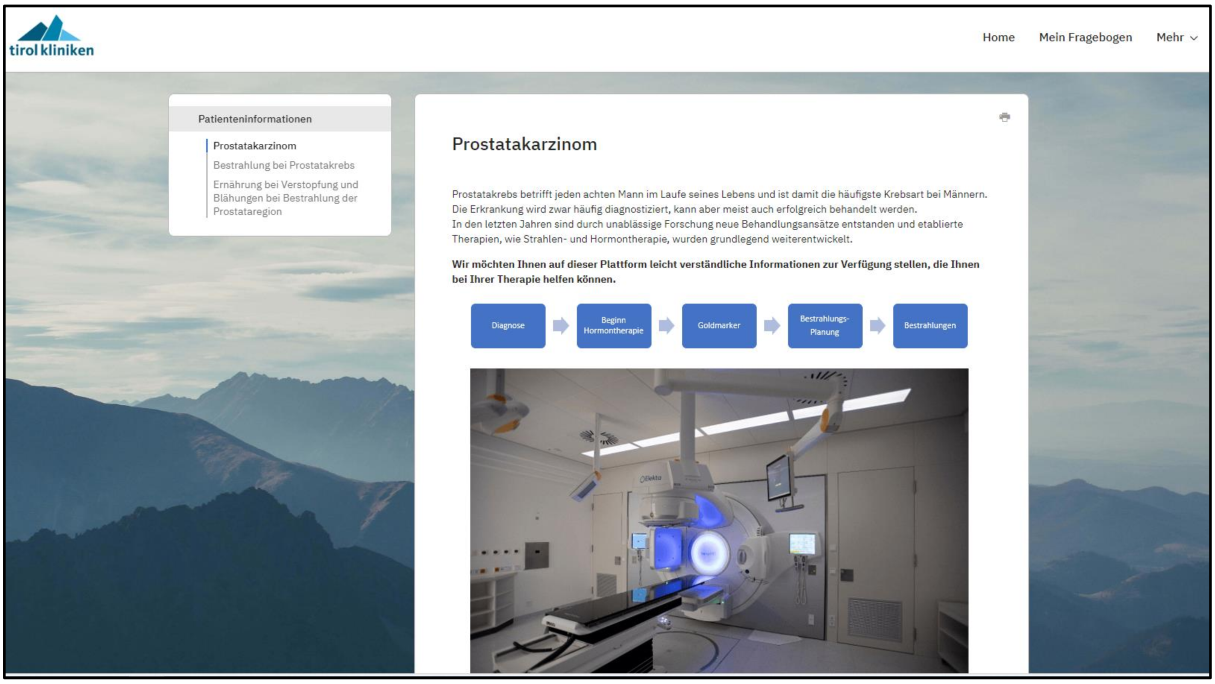

Screenshot 3. This screenshot is an example of the disease and treatment specific information, which is shown in the patient portal.

Supplementary Materials 3: Questionnaires used and item and threshold selection process

Selection Process

The process of selecting the questionnaires and their respective items was conducted in a multi-round, unstandardized consensus process by an expert panel consisting of five doctors and two PRO experts. The primary goal was to identify questionnaires that were both clinically relevant and widely recognized in research and clinical practice. This approach ensured the assessment focused on the outcomes most relevant to patients and healthcare providers, facilitating informed clinical decision-making.

Questionnaires and Items

1. **EORTC QLQ-C30**: Selected for its robust validation and comprehensive coverage of cancer-related quality of life aspects.
2. **EPIC-26**: Chosen for its specific focus on prostate cancer-related symptoms and its frequent use in prostate cancer research.
3. **Hornheider Screening Instrument**: Used at baseline to screen for psycho-oncological support needs, with a score of >4 indicating the need for such support.

Thresholds for Interpretation

- **EORTC QLQ-C30**: Utilized validated thresholds available in the literature to interpret the results. (https://pubmed.ncbi.nlm.nih.gov/31639445/)
- **Hornheider Screening Instrument**: A score above 4 was used as the threshold for indicating psycho-oncological support needs (https://pubmed.ncbi.nlm.nih.gov/12079234/).
- **EPIC 26 and items from the EORTC Item Library**: For items without predefined thresholds, the expert panel defined clinically relevant thresholds, which were then integrated into the ePRO system.

Visualization and Implementation

The expert panel also determined how the PROM results should be visualized for both healthcare providers and patients. This involved setting appropriate thresholds to trigger clinical actions, thereby ensuring the ePRO system's utility in routine clinical practice. The system was tailored to fit local

workflows through focus groups, ensuring it met clinical needs and was easy to integrate into daily practice.

Baseline and Follow-up Assessments

- **Baseline**: Full versions of QLQ-C30, EPIC-26, and Hornheider Screening Instrument.
- **Monitoring During Radiation therapy**: Selected items from QLQ-C30 and EORTC item library, focusing on radiotherapy-associated symptoms.
- **Follow-up**: Full versions of QLQ-C30 and EPIC-26, with additional questions regarding androgen deprivation therapy (ADT) and regular urology appointments.

Supplementary Material 4: Feasibility baseline

Variable	N = 40[1]
Did respondent have issues logging into the portal?	
No	31 (77.5%)
Yes	9 (22.5%)
Did filling out the questionnaires work?	
Yes	38 (95.0%)
No	2 (5.0%)
Were you able to navigate the questionnaire? (forward, back, finish)	
Yes	33 (82.5%)
No	6 (15.0%)
Missing	1 (2.5%)
Were there any technical difficulties?	
No	35 (87.5%)
Yes	5 (12.5%)
Would you like to see your own test results?	
Yes	28 (70.0%)
No	12 (30.0%)

[1]n (%)

Supplementary Materials 5: Health care professional survey complete results

	Professional group		
Variable	Medical and Psycho-oncology (n = 6)	Administration (n = 2)	Total (N = 8)
Years of professional experience			
>10	3 (50.0%)	1 (50.0%)	4 (50.0%)
0-3	2 (33.3%)	0 (0.0%)	2 (25.0%)
3-10	1 (16.7%)	1 (50.0%)	2 (25.0%)
The training for my group was understandable and sufficient			
Very much	4 (66.6%)	2 (100%)	6 (75.0%)
Quite a bit	1 (16.7%)	0 (0.0%)	1 (12.5%)
Did not receive any training	1 (16.7%)	0 (0.0%)	1 (12.5%)
Does not apply	0 (0.0%)	0 (0.0%)	0 (0.0%)
A little bit	0 (0.0%)	0 (0.0%)	0 (0.0%)
I had to answer questions from patients about the application			
<5%	3 (50.0%)	1 (50.0%)	4 (50.0%)
10-25%	2 (33.3%)	1 (50.0%)	3 (37.5%)
25-50%	1 (16.7%)	0 (0.0%)	1 (12.5%)
>50%	0 (0.0%)	0 (0.0%)	0 (0.0%)
Additional workload? Question only for Administration			
5-20 min/day	-	2 (100%)	2 (100%)
<5 min/day	-	0 (0.0%)	0 (0.0%)
>20 min/day	-	0 (0.0%)	0 (0.0%)
Would you recommend it to other health care professionals?			
Very much	4 (66.6%)	0 (0.0%)	4 (50.0%)
Quite a bit	1 (16.7%)	1 (50.0%)	2 (25.0%)
A little bit	1 (16.7%)	1 (50.0%)	2 (25.0%)
Not at all	0 (0.0%)	0 (0.0%)	0 (0.0%)

Supplementary Materials 6: Patient acceptability questionnaires [original in German]

Baseline:

1. Did you have difficulties logging in to the portal? ☐ Yes ☐ No

2. Were you able to complete the questionnaires? ☐ Yes ☐ No

3. Were you able to navigate within the questionnaire (forward, back, finish)? ☐ Yes ☐ No

4. Where there any technical difficulties? ☐ Yes ☐ No

5. Would you like to see your own results after completing the questionnaire? ☐ Yes ☐ No

6. Do you have any further comments (suggestions, wishes, missing items, etc.)?

End of Treatment:

1. Were all questionnaires comprehensible? ☐ Yes ☐ No

2. Would it be okay for you to complete the questionnaires regularly as part of your clinical care? ☐ Yes ☐ No

3. Did you need help filling out the questionnaires? ☐ Yes ☐ No

4. Was the completion of the questionnaires an additional burden? ☐ Yes ☐ No

5. Did you encounter any barriers to completing the questionnaires ☐ Yes ☐ No

6. Do you have any further comments (suggestions, wishes, missing items, etc.)?

Patients were asked to specify any difficulties (e.g. when logging in to the portal) or help they had.

Supplementary Materials 7: Onboarding process

The ePRO program was introduced by two patient-reported outcome (PRO) experts (BH, JL) through an initial session, which detailed the program's features and benefits. This presentation was followed by small standard operating procedure documents (created by SV) tailored to the needs of different user groups.

Administrative Staff: Guidance on creating patient accounts and printing login data.

Physicians: Instructions on accessing patient lists and navigating individual reports

Psycho-Oncologists: Specific directions on leveraging the platform for psychological assessments.

To integrate the ePRO program seamlessly into clinical workflows, a direct link to the web-based platform was installed within the clinic's software. A designated physician (SV) served as the internal expert for the program, acting as the primary contact for troubleshooting and addressing emerging issues. To improve awareness and utilization of the ePRO platform, the PROs of individual patients were repeatedly discussed in the clinic's team meetings at the beginning.
